# Associations between gene polymorphisms and selected meat traits in cattle — A review

**DOI:** 10.5713/ab.20.0672

**Published:** 2021-01-04

**Authors:** Magdalena Zalewska, Kamila Puppel, Tomasz Sakowski

**Affiliations:** 1 Department of Bacterial Physiology, Institute of Microbiology, Faculty of Biology, University of Warsaw, Warsaw 02-096, Poland; 2 Institute of Animal Science, Warsaw University of Life Sciences, Warsaw 02-786, Poland; 3 Department of Biotechnology and Nutrigenomics, Institute of Genetics and Animal Biotechnology, Polish Academy of Sciences (PAS), Magdalenka 05-552, Poland

**Keywords:** Beef, Carcass Quality, Cattle, Marker-Assisted Selection, Meat Trait, Single Nucleotide Polymorphism

## Abstract

Maintaining a high level of beef consumption requires paying attention not only to quantitative traits but also to the quality and dietary properties of meat. Growing consumer demands do not leave producers many options for how animals are selected for breeding and animal keeping. Meat and carcass fatness quality traits, which are influenced by multiple genes, are economically important in beef cattle breeding programs. The recent availability of genome sequencing methods and many previously identified molecular markers offer new opportunities for animal breeding, including the use of molecular information in selection programs. Many gene polymorphisms have thus far been analyzed and evaluated as potential candidates for molecular markers of meat quality traits. Knowledge of these markers can be further applied to breeding programs through marker-assisted selection. In this literature review, we discuss the most promising and well-described candidates and their associations with selected beef production traits.

## INTRODUCTION

Carcass traits, alongside milk production traits, are the essential traits in the cattle industry. Optimizing beef production is a very complicated, time-consuming, and labor-intensive process. Thus, progress in this field is possible due to the accuracy of animal breeding value assessments. In classic genetic improvement programs, selecting animals with desired properties is based on observed phenotypes, with knowledge only on animal pedigree. Molecular genomics methods allow the identification of potential candidate genes that control economically important traits in beef production. By applying genomic techniques to breeding programs, genomic progress may be achieved faster. Classic breeding methods assess the value of breeding cattle based on the production characteristics of their offspring. However, these methods are more time-consuming and labor-intensive than analyzing an animal’s genotype for selected markers, which would indicate the defined genetic predisposition of that single animal.

Muscle tissue after slaughter undergoes various biophysical and chemical changes and events that convert it into meat, and these may vary with time, temperature, individual genotype, and individual predispositions, as well as sex, age, muscle type, or species of the animal [1,2]. Beef carcass quality in particular strongly depends on these elements. The abovementioned factors affecting meat quality can be divided, in general, into two groups: genetic factors (e.g., breed or genotype) and management systems (e.g., feeding, handling details, or slaughtering conditions). Meat quality traits are difficult to improve by traditional selection because most related features are assessed after passing several generations of animals. Different genomics techniques offer outstanding opportunities to enhance the genetic potential of food-producing animals and implement this potential in breeding programs through marker-assisted selection (MAS) [3].

Typical meat quality parameters include color, intramuscular fat content, marbling score (MBS), and water-holding capacity. Meat with a bright red color may increase consumer satisfaction and thereby influences purchasing decisions. The ability to predict meat tenderness is an essential issue facing the beef industry because meat tenderization during the post-mortem period is highly variable among carcasses, and consumers demand a consistent product [4]. By offering meat products with guaranteed quality, consumers may increase their beef consumption and be more willing to pay a higher price [5]. A high degree of marbling improves meat quality in terms of juiciness, flavor, and tenderness (shear force). MBS has also been recognized as an essential factor determining the economic value of beef [6,7]. Water-holding capacity is economically and technologically critical not only for consumer choice in the store and appeal when cooking but also for the meat processing industry [8]. In addition, carcass quality and backfat thickness are strongly associated with the percentage of products sold, and in turn, the weight of retail cuts is related to the rib eye area. Fat deposition, especially intramuscular fat, can interfere with meat tenderness perception [5]. Moreover, intramuscular fat represents a vital beef quality trait, as it contributes to the juiciness, tenderness, and flavor of cooked meat [9]. Beef quality depends not only on MBS but also on fatty acid (FA) composition, which determines the quality of the fat. Thus, the intramuscular FA profile of cattle has become an essential issue in the beef industry. The high abundance of monounsaturated fatty acid (MUFA) content in muscle exerts its effect at lower melting points, which in turn has a positive effect on beef flavor and tenderness [10]. One of the most critical MUFAs in beef fat is oleic acid (C18:1), whose content is the most important for controlling beef flavor. Moreover, the higher unsaturated FA content is associated with juicer meat [4], and it is considered to be the primary source of the aroma of the cooked meat [11]. Red meat is a rich source of high-quality protein and provides a variety of essential nutrients, as well as saturated fatty acids (SFAs). The microorganisms within the rumen of cattle hydrogenate a majority of dietary unsaturated FAs, which results in a higher concentration of SFAs in beef compared to meat from non-ruminant animals. Thus, one of the significant concerns that affect beef intake is the SFA content in meat. For many years, dieticians and physicians have recommended reducing the consumption of foods rich in SFAs or even completely eliminating them from the diet, with a particular emphasis placed on beef [12]. Beef consumption in the US has decreased from 40.4 kg/person in 1976 to 29.5 kg/person in 2003 [11]. Although this notion has been challenged by recent evidence, certain SFAs, particularly lauric (C12:0), myristic (C14:0), and palmitic (C16:0) acids, are often associated with cardiovascular diseases [13]. Juicier meat has been reported to show higher unsaturated FA content and higher beef quality [10]. In summary, fat content and FA profiles are fundamental parameters for meat producers and consumers [14].

The single nucleotide polymorphism (SNP) is the most common form of DNA variation in mammals. SNPs are abundant, normally biallelic, and easy to detect by automatic techniques. It has been reported that SNPs in some genes are associated with beef tenderness (e.g., calpain-1 and calpastatin) or MBS (e.g., leptin, diacylglycerol acetyltransferase, retinoic acid receptor-related orphan receptor C, bovine growth hormone (GH), and stearoyl-CoA desaturase) [6] (Table 1). Many of these associations have been reported in independent studies, although some of them have failed to be confirmed due to inadequacies in the experimental design, primarily due to sample sizes being too small. Sample sizes need to be large enough to ensure that this sort of error is minimized [15].

In this study, we focused on the most promising and well-described candidates and their associations with selected beef production traits (Table 2).

### Leptin

Leptin (Lep) is a hormone (similar to cytokines) secreted by adipose tissue and can also be found in gastric epithelial cells. It is involved in the homeostatic system responsible for the assimilation, storage, and use of energy from nutrients. Thus, it is considered to be associated with carcass fat, body weight, and growth rate in cattle [16]. Lep exerts acute effects on metabolism, as well as on long-term body weight regulation [17,18]. The economic importance of factors related to appetite, such as feed intake, body weight gain, and fat deposition, has led to studies of the effect of leptin on carcass characteristics in beef cattle [19]. Research conducted on purebred Aberdeen Angus sires and dams (a mixture of purebreds of various breeds and crossbreeds including Aberdeen Angus, Aberdeen Angus-crossbreed, Simmental-crossbreed, and Limousin-crossbreed) showed the impact of the leptin gene’s UASMS2 SNP on meat flavor. Animals with the TT genotype had a higher liking score than animals exhibiting CC or CT genotypes. Moreover, a strong association was observed between this SNP and sirloin fat thickness: animals with the CC genotype had less fat surrounding the sirloin than animals with the CT or TT genotype [16]. This SNP is also known to be associated with backfat thickness, as well as MBS, with TT animals having a higher value for these trait [20]. However, the research conducted by Curi et al [21] on purebred *Bos indicus* animals and crossbreeds of *Bos indicus* and *Bos taurus* did not show a significant effect of the Lep/BsaAI (Y_11369.1:g.1620G>A) SNP on backfat thickness, rib eye area, intramuscular fat, shear force, or myofibrillar fragmentation index. Shin and Chung [22] reported, in Korean Native steers, a significant effect of the C1180T SNP on backfat thickness and MBS, with the C allele being associated with increased values for both traits. However, research conducted on Angus, Hereford, Limousin, Simmental-type, Charolais cattle, as well as on various small breeds, regarding the Lep SNP described initially by Buchanan et al [23] (exon 2, arginine to cytosine substitution) did not reveal differences in feed intake, feed efficacy, or feeding behavior among animals with different leptin genotypes. Moreover, slaughter weight, carcass weight, and carcass MBS did not differ between animals of different genotypes, though animals with the TT genotype had more carcass grade fat compared to heterozygotes and CC homozygotes, and thymine homozygotes had lower meat yield compared to CT and CC genotype animals. Furthermore, this research was not able to show an association between animal genotype and body composition, distribution of carcass fat, or total carcass lean [24]. In addition, research conducted by He et al [25] did not revealed an association of described leptin genotype with growth performance, adipocyte cellularity, meat quality, and FA profile in beef steers depending on the diet. Lastly, an analysis conducted on purebred Aberdeen Angus, Belgian Blue, Blonde d’Aquitaine, Charolais, Friesian, Hereford, Limousin, Salers, and Simmental cattle showed no correlations for four SNPs (UASMS1, UASMS2, a SNP within exon 2 described initially by Buchanan et al [23], and a SNP within exon 3 described by Haegeman et al [26]) with regard to genotypes when considering the trait of intramuscular fat [9].

### Thyroglobulin

Thyroglobulin (TG) is a precursor of thyroid hormone that plays an essential role regulating metabolism and fat deposition homeostasis. Moreover, TG works as a carrier for triiodothyronine and thyroxin, which participate in fat cell development (both growth and differentiation) [27]. Different genetic variations occurring in the 5′ region of the *TG* gene promoter are currently widely used in MAS-based breeding programs to improve prediction of MBS and beef quality [28]. The 5′ flanking region of the *TG* gene plays a crucial role in transcriptional regulation, thus different mutations within its structure affect the different affinities of the transcriptional factors [29]. However, some studies have indicated that this particular SNP occurs in monomeric form among different populations, such as native Bali cattle, and Angus or Hereford [30,31]. The C422T SNP of the *TG* gene has been recognized as being associated with MBS and fat deposition in beef, as well as fat thickness, total lipids, ribeye area, and shear force. Animals with the TT genotype have a higher MBS than animals exhibiting the CC and CT genotypes. This research was conducted on Nellore, RubiaGallega ×Nellore, Canchim, and three-way cross Brangus and Simmental-type cattle [32]. Moreover, Gan et al [28] have found significant SNPs in the 3′ flanking region of the gene. This study, conducted on Simmental, Angus, Hereford, Charolais, Limousin, Qinchuan, Luxi, and Jinnan cattle, have found four SNPs (G133C, G156A, C220T, and A506C) correlated with MBS. Moreover, an analysis conducted on Korean Native cattle (steers with a known pedigree) revealed a significant association between the C422T SNP and MBS. Animals exhibiting the CC and CT genotypes had a higher MBS than animals with the TT genotype. Additional associations between other identified SNPs (C257T, A335G, and C422T) and analyzed meat parameters were detected [27]. Moreover, research carried out on large populations of purebred Angus and Shorthorn cattle, as well as crossbreeds (including Angus, Shorthorn, Charolais, Shaver, Limousin, Simmental, Santa Gertrudis, and Red Composite) revealed a strong relationship between TG5 SNPs and MBS [33]. An analysis conducted on Simmental, Angus, Hereford, Charolais, Limousin, Qinchuan, Luxi, and Jinnan cattle indicated that of the SNPs in the 3′ flanking region of the *TG* gene, T354C, G392A, A430G, and T433G were significantly associated with beef MBS. For all of these SNPs, the animals exhibiting the CC genotype had higher value for this trait than those with CT or TT genotypes [34]. A study conducted by Bennett et al [35] on the Meat Animal Research Center population (0.25 Angus, 0.25 Hereford, 0.25 Gelbvieh, and 0.25 Simmental) described the influence of a TG SNP on meat tenderness, with CT animals having more tender meat than CC and TT ones, which indicated a heterozygote advantage for the tenderness trait. In a population of Chinese cattle (including Angus, Charolais, Luxi, Qinchuan, Hereford, Limousin, Simmental, and Jinnan) SNPs in the 5′ flanking region of the *TG* gene (G275A, G277C, G280A, and C281G) were strongly associated with average daily weight gain, but not with live weight, carcass weight, MBS, loin muscle area, or backfat thickness [29]. Moreover, the research conducted by Casas et al [36] on a very diverse cattle population (Angus, Hereford, Norwegian Red, Swedish Red and White, Wagyu, Friesian, and MARC III [0.25 Angus, 0.25 Hereford, 0.25 Red Poll, and 0.25 Pinzgauer]), which aimed to assess the effect of the C422T on carcass traits in beef cattle, revealed a strong association of this SNP with MBS only in the Wagyu population.

### Calpain

The calpain/calpastatin system is an endogenous, calcium-dependent proteinase system responsible for mediating the proteolysis of essential myofibrillar proteins during the post-mortem storage of carcasses and cut meats at refrigerated temperatures [37]. The calpain system is responsible for the proteolysis of cytoskeletal proteins and intermediate filaments during aging. It comprises endogenous proteases (calpains), which are considered the primary candidates for muscle protein degradation initiated during the first 24 hour post-mortem, as well as their inhibitor, calpastatin [2]. Meat tenderness largely depends on the overall integrity of muscle cells, which can be disrupted by the degradation of crucial myofibrillar and cytoskeletal proteins. The most important process for maximizing meat tenderness is the appropriate activation of the endogenous proteolytic enzymes (i.e., calpains, cathepsins, and caspases) responsible for degrading muscle fibers, but temperature, time of ageing and pH are also crucial. The *CAPN1*, the most critical gene in meat tenderization, encodes the enzyme calpain-1. Calpain-1 is responsible for the digestion of desmin and costamere structures during the meat aging process. It has been reported that the CAPN1: c.947G>C SNP (with a substitution from alanine to glycine) is associated with meat tenderness in Angus and Belmont Red cattle and that the same association exists for the g.6546C> T SNP in Brahman, Santa Gertrudis, and Belmont Red cattle; however, no purebred European cattle exhibit this association with the g.6546C>T SNP [38]. Research conducted on a commercial breed of Swedish cattle regarding this SNP showed that meat samples obtained from animals with the CC genotype were more tender than meat samples from animals with the GG genotype. The heterozygote produced meat with a tenderness score between that of homozygotes [1]. Furthermore, study on the C6545T (substitution from cytosine to thymine) of Casas et al [39] showed associations between this SNP and tenderness for *Bos taurus* (lower values for homozygotic animals, p<0.1, trend level) and *Bos taurus*× *Bos indicus* populations (lower values also for homozygotic ones; p<0.001), as well as flavor for a *Bos taurus*×*Bos indicus* population (higher values for homozygotic animals, p<0.005). Zhang and Li [40] conducted research on Nanyang, a Chinese breed of cattle, aiming to assess the relationship between the calpain-2/HhaI SNP and growth traits, such as body weight, withers height, body length, and heart girth. The study revealed many differences depending on animal genotype; for example, for body weight, a higher value was observed for animals exhibiting heterozygosity than homozygosity (p<0.05) (after six months); for wither height, highly homozygotic animals were smaller than heterozygotic ones (p<0.05) (after six months). Moreover, other analyses have revealed a significant influence of analyzed SNPs within the calpain gene on meat traits, including juiciness, flavor, and overall liking, for breeds belonging to both *Bos taurus* and *Bos indicus* (e.g., Angus and Brahman), as described by Robinson et al [41]. Lastly, studies of Sun et al [42] have found strong association between CAPN1 A4558G genotypes with meat tenderness (with lower value for GG homozygote), MBS (with lower value for GG animals), and no association of these gene with meat and fat color in Chinese Simmental cattle. During the same studies the effect of CAPN1 C4684T on meat tenderness (higher value for CC animals compared to TT ones, and for CT animals compared to TT ones) was stated.

A study on Korean Native cattle did not show any associations between twelve newly identified SNPs and cold carcass weight, though it did reveal an association (p<0.001) between the c.2151*479C>T SNP located in the 3′ untranslated region (UTR) of *CAPN1*, with MBS having a significantly higher value for animals with the CC genotype [43]. Moreover, an association study on a commercial cow population in Ireland (beef×dairy crossbreeds) found an association between capnI/BtgI (SNP located within exon 9; alanine to glycine substitution) and shear force (p<0.05), with a higher value for GG genotypes [44]. Similar results were found in a study on a large group of commercial cattle in the United States [45]. Moreover, research conducted on a mixed cattle group consisting of Nellore, Angus×Nellore, Canchim, Brangus, and Brown Swiss on breeds with regard to capn4751 (intron 7; cysteine to thymine substitution) did reveal differences among the animals exhibiting different genotypes for shear force and myofibrillar fragmentation index (with a higher value for TT genotypes and a higher value for CT genotypes, respectively) [46]. Another analysis conducted by Curi et al [47] with a similar group of animals but a different SNP (CAPN316, g.5709C>G located at exon 9 of the *capnI* gene) found associations with shear force (with a higher value for GG animals [p<0.05]) and with the myofibrillar fragmentation index (with higher values for GG animals [p<0.05]).

### Calpastatin

Calpastatin (CAST), together with calpain, participates in the proteolysis of myofibrillar proteins during the post-mortem storage. It inhibits μ- and m-calpain activity and, therefore, regulates post-mortem proteolysis. Moreover, CAST activity has been correlated with reduced meat tenderness [37]. Research conducted on *Bos taurus*, *Bos taurus*×*Bos indicus*, and *Bos indicus* populations analyzing the A2959G (in the 3′ UTR region of the gene; effects in guanine to adenine substitution (United States Patent No. US7625698B2) [48] revealed a strong association of this SNP with shear force at 14 days post-mortem for the *Bos taurus* and *Bos taurus*×*Bos indicus* populations (both with lower values for heterozygotic animals). Similar associations have been also found with regard to: tenderness for the B*os taurus* and *Bos taurus*×*Bos indicus* populations (with lower values for homozygotic animals); juiciness for the *Bos taurus* population (with lower values for homozygotic animals); and flavor for the *Bos taurus*×*Bos indicus* population (with higher values for heterozygotic animals) [39]. In addition, the research conducted by Robinson et al [41] revealed the effect of the CAST SNPs described by Barendse (United States Patent No. US7625698B2) [48] on all traits analyzed by sensory panels. The most significant associations of analyzed SNPs were found for the tenderness of Achilles-hung striploins, but also for tenderstretched striploin and rump steaks. The study conducted on Chinese Simmental cattle have analyzed the effect of CAST T596C SNP on meat tenderness, MBS, meat color, and fat color, but researchers did not shown associations [25]. A study conducted on Nellore cattle and their crosses with *Bos taurus* to determine the effect of the g.98535683A>G SNP on rib eye area, backfat thickness, total lipids, shear force, and myofibrillar fragmentation index showed an association of the genotypes with shear force and myofibrillar fragmentation index, with the homozygous AA genotype being more favorable than the AG genotype. The lack of other associations between the allelic forms of the *CAST* gene and traits related to growth (i.e., rib eye area) and fat deposition (i.e., backfat thickness and total lipids) is understandable, as calpastatin does not appear to participate in the physiology of these traits [49]. Moreover, research conducted on a mixed population (Nellore, Angus×Nellore, Canchim, Brangus, and Branviech) with regard to the CAST g.2959A>G SNP (with a restriction site for DdeI in 3′UTR; effects in arginine to guanine substitution) did reveal differences among the genotypes for shear force and myofibrillar fragmentation index (with higher values for both AG and AA genotypes) [46]. Conversely, a study by Curi et al [47] with a similar group of animals but a different SNP (g.282C>G, restriction site for RsaI; SNP located within intron 5 of the calpastatin gene) did not show such associations.

### Stearoyl-CoA desaturase

Stearoyl-CoA desaturase (SCD) is an enzyme participating in the conversion of SFAs into unsaturated FAs in mammalian adipocytes (by introducing a double bond). For ruminants, FAs delivered with fodder are processed by microorganisms in rumen and are adsorbed as SFAs. It is primarily determined by key lipogenic enzymes in FA synthesis pathways [13]. The composition of stored FAs depends on SCD action, among other factors [50]. Research conducted on Japanese Black cattle revealed eight SNPs in the full-length cDNA of *SCD* gene, of which three were located within the 5′ region. The T878C SNP causes a substitution of valine to alanine in the coded protein, and this change has been associated with a higher MUFA percentage in intramuscular fat [50]. A study conducted on Fleckvieh cattle (a Simmental cattle type dual-purpose breed) revealed a significant association of this SNP with FA composition [51]. In addition, a study conducted on a Canadian population of commercial crossbred beef steers detected significant additive or dominance effects for SFAs, MUFAs, conjugated linoleic acids, and polyunsaturated FAs. Moreover, CC genotypes are associated with lower concentrations of SFAs, higher concentrations of MUFAs, and a higher concentration of polyunsaturated fatty acids (PUFAs) compared to TT genotypes [52]. However, previous research on Spanish commercial beef cattle (crossbred between Retinta and Continental cattle) did not reveal an association between the T878C SNP and backfat or intramuscular fat content [53]. This result differs from the data obtained from a Chinese Simmental breed analysis conducted by Wu et al [54]. Instead, they found associations between T878C and intramuscular fat content and shear force value, which are consistent with the findings of Taniguchi et al [50] in Japanese Black cattle. Moreover, an association study was performed on a Wagyu×Limousin crossbreed [55]. In this study, three SNPs were identified (g.4706C>T, g.7534G>A, and g.7864C >T). It has been presented that SNPs within this gene structure are positively associated with skeletal muscle fat deposition and FA composition: g.4706C>T is associated with the relative amounts of SFAs and MUFAs; g.7534G>A with beef MBS (which is higher in GG than in CC animals) and the relative amount of SFAs; and g.7864C>T with the relative amounts of SFAs and MUFAs (which are higher in CC than in TT animals) and conjugated linoleic acids. Moreover, it has been recognized that all three SNPs are associated with estimated SCD activity [55]. Lastly, studies conducted on Wagyu cattle showed the gene effects on luster, firmness, and texture of the meat were detected (though none remained significant after multiple testing corrections) [11].

### Diacylglycerol acetyltransferase

Diacylglycerol acetyltransferase (DGAT1) is considered a crucial microsomal enzyme that catalyzes the last step of the synthesis of triglycerides from diacylglycerol and FAs in triglyceride synthesis [56]. The K232A SNP is an AA/GC dinucleotide substitution that affects a lysine to alanine amino acid substitution. Studies conducted on Aberdeen Angus purebred sires and dams that were a mixture of purebreds of various breeds and crossbreeds, including Aberdeen Angus, Aberdeen Angus-crossbreed, Simmental-crossbreed, and Limousin-crossbreed revealed associations of this SNP with sirloin weight after maturation and sirloin fat depth. Moreover, an increase in sirloin weight was positively correlated with an increase in the fat surrounding muscle [16]. In a study conducted on Simmental, Hereford, Limousin, Angus, Charolais, Luxi, Qinchuan, and Jinnan cattle revealed significant associations between the c.572A>G SNP and backfat thickness, MBS, fat color, and shear force: animals with the BB genotype had higher values for backfat thickness and lower values for MBS, fat color, and shear force than animals with the AA genotype. This study showed strong associations between the c.1416T>G SNP and backfat thickness, MBS, fat color, and shear force, with higher values for backfat thickness and lower values for longissimus muscle area, fat color, and shear force for FF genotype animals than for EE genotype animals [56]. Curi et al [21] conducted a study on purebred Nellore and Canchim cattle, as well as crossbreeds between Nellore and Angus, RubiaGallega, Brangus, and Simmental-type cattle, which showed a significant correlation between the K232A SNP and backfat thickness. However, they did not find any effects on studied traits. A study conducted within a population of Slovak Pinzgauer steers regarding K232A showed that the AA genotype was favorable for all of analyzed traits [57]. Lastly, a study conducted on Polish Holstein bulls revealed a significant effect of the K232A on C12:0 content and conjugated linoleic acids [14].

### Bovine growth hormone and bovine growth hormone receptor

The GH interacts with the growth hormone receptor (GHR) and thus affects growth and metabolism. Alterations in the functional region of the *GHR* gene can change the binding and signaling pathways of GH, and subsequently, the activity of GH in tissue [58]. GH and GHR have been associated with drip loss, body weight, and MBS. Studies conducted on GH1: c.457C>G showed that this SNP is significantly associated with rump fat and with eye muscle area and carcass weight, but only at the trend level. Animals exhibiting the CC homozygous genotype had higher fat thickness [59]. An association study performed on commercial Japanese Black Cattle (Wagyu) with regard to FA composition of the longissimus thoracis muscle and carcass traits showed a connection between L127V (SNP effects in the leucine to valine substitution) and subcutaneous fat thickness, and between T172M (SNP effects in the threonine to valine methionine) and carcass weight, rib thickness, subcutaneous fat thickness, and firmness [11]. Moreover, GHR SNP may affect meat quality traits. Stasio et al [58] analyzed the SNP (A257G) in the cytoplasmic domain of the *GHR* gene (a arginine to glycine substitution) in Piedmontese cattle with regard to growth, size, and meat conformation.

### Fatty acid binding protein 4

The fatty acid-binding protein 4 (*FABP4*) gene plays an essential role in lipid hydrolysis and intracellular FA trafficking in different tissues. The FAs are essential as signaling molecules as their intracellular uptake triggers preadipocyte differentiation and terminal differentiation-related gene expression [60]. Significant functions of FABP4 include FA uptake, transport, and metabolism [13]. The association of c.220A>G (I74V) with palmitoleic acid in the adipose tissue of beef cattle has been described in Japanese Black cattle crossbreeds. The I/I homozygote exhibited a higher percentage (0.5%) of C16:1 than the V/V homozygote (p<0.05) [61]. Moreover, a study on Holstein cattle revealed a 1.5% higher percentage of C16:0 in animals exhibiting V/V genotypes than I/V genotypes at the same SNP [62]. Both groups did not find any significant associations between the analyzed SNPs and any carcass traits. In addition, the research conducted by Curi et al [21] on purebred *Bos indicus* animals and *Bos indicus* and *Bos taurus* crossbreeds did not show a significant effect of the FABP4/MspI SNP on backfat thickness, rib eye area, intramuscular fat, shear force, or myofibrillar fragmentation index. However, studies on Korean Native cattle conducted by Cho et al [63] showed associations of two SNPs—c.220A>G (I74V) and c.328G>A (V110M)—with backfat thickness and carcass weight (both with higher scores for heterozygotes). Moreover, research conducted by Oh et al [10] revealed strong associations between analyzed FABP4 SNP and carcass traits: the c.280A>G with backfat thickness, the c388G>A with MBS), and also c.280A>G, c.388G>A, c.408G>C, c.456A>G with FA composition (higher percentage of C18:1). In addition, associations between the c.388G>A, c.408G>C, and c.456A>G SNPs and MUFA percentage were found (with higher values for GG animals, for CC animals, and for AA and AG animals, respectively) [10]. Moreover, it has been shown that FABP4 genotypes may affect MBS and subcutaneous fat depth in Wagyu and Limousin animals [64].

### Stearol regulatory element-binding protein 1

Stearol regulatory element-binding protein 1 (SREBP) is a key transcriptional activator of several lipogenic genes in adipose tissue [51]. Matsuhashi et al [11] analyzed a population of commercially available Japanese Wagyu cattle for associations of a previously described SNP in SREBP-1 (an84-bp insertion [allele L] or deletion [allele S]); they found an association of this SNP with FA composition of muscle (higher values for LL and LS genotypes for tetradecanoic acid C14:0 and hexadecanoic C16:0 and with carcass weight with lower values for LL and LS animals in Japanese Black cattle [65] and FA composition of muscle (a significant associations with the concentration of stearic (C18:0), linoleic (C18:2) and PUFA for Korean Hanwoo was stated [66]. Moreover, Bartoň et al [51] assessed the association between this SNP and the FA profile of beef muscle and subcutaneous fat in Fleckvieh bulls and found that it was associated only with tetradecenoic acid (C14:1 cis-9) content.

### Fatty acid synthase

Fatty acid synthase (FASN) is a multifunctional enzyme complex that plays an essential role in determining the FA profile of ruminant tissues. It is mainly involved in the MUFA synthesis pathway and incorporation into triacylglycerols and phospholipids [52]. The thioesterase domain within FASN participates in terminating FA synthesis and releasing synthesized SFAs. The thioesterase domain of FASN, therefore, plays a crucial role in determining chain length. It was hypothesized that variation in this domain among animals could account for heritable differences in FA composition [12]. Matsuhashi et al [11] analyzed a population of commercially available Japanese Wagyu cattle, searching for associations of two previously described SNPs in FASN: g.16024A>G (T1950A) and g.16039T>C (W1955R). They did not find any associations with meat yield traits, such as carcass weight, rib eye area, rib thickness, subcutaneous fat thickness, or carcass yield estimate, or with meat quality traits, such as beef MBS, beef color standard, luster, firmness, texture, moisture, or crude fat. Furthermore, a study on a Holstein population by Narukami et al [62] also did not reveal any significant correlations between the analyzed SNP A5855G (with the amino acid substitution T1952A) and carcass traits or FA composition. However, other researchers have found associations of several SNPs in FASN with meat quality traits. A study conducted on crossbred Japanese Black and Limousin (F2 individuals) cattle showed two non-synonymous mutations—g.16024A>G (a threonine [T] to alanine [A] substitution) and g.16039T>C (a tryptophan to arginine substitution)—within 34 exons of the gene. Two genotypes (TW and AR) had a significant effect on the FA composition of backfat, intermuscular fat, and intramuscular fat. The TW haplotype was associated with increased C18:0 and C18:1 content and the ratio of monounsaturated to saturated FAs, as well as decreased C14:0, C14:1, C16:0, and C16:1 content [67]. Moreover, Li et al [52], who were studying Canadian commercial crossbreed steers, found associations between the g.17924A>G SNP (amino acid substitution from threonine to alanine) and various traits: strong associations were found for a wide range of SFAs, several MUFAs, and a single long-chain polyunsaturated FA. In particular, the animals exhibiting the AA genotype had higher concentrations of SFA 14:0 and lower concentrations of oleic acid (9c-18:1). The research conducted by Zhang et al [12] on purebred American Angus cattle revealed that the g.17924A>G SNP was significantly associated with the FA composition of muscle. Cattle with the GG genotype had a lower C14:0 level, C16:0 level, and total SFA content, as well as a higher health index, C18:1 content, and total MUFA concentration in the total lipids and triacylglycerol fraction than animals with the AA genotype [12].

## CONCERNS OF BEEF INDUSTRY

Beef consumers increasingly seek meat of high and consistent quality. Beef quality includes sensory quality traits (tenderness, flavor, juiciness, color, etc.), nutritional value, and healthiness, and also takes other issues into account, such as animal welfare, environmental concerns, traceability [68]. Whereas the latter are more complex or subjective, the former is directly associated with the muscle biology traits of the animals during post-mortem processing treatment [15]. The identification of genetic markers that are positively associated with economically important traits in livestock has the potential to significantly alter the rate of genetic improvement through the use of MAS-based breeding programs. Genomic studies of production traits can be applied to MAS to increase the frequency of favorable alleles in the target population [27]. In agriculture and animal science, the outcome of genomics includes improvements in food safety, traceability, and the quality of animal products through increased breeding efficiency and better knowledge of animal physiology. DNA-based techniques have been developed for the detection of bacterial contamination (beef safety), for species and animal identification (breed and individual traceability), and for genetic selection (genetic improvement of beef quality) [69,70]. MAS allows for the accurate selection of specific DNA variations that have been associated with desired performance traits. This knowledge could be used to increase the frequency of particular markers that are positively associated with a trait by selecting animals that carry those variations [71]. The implementation of MAS required careful consideration of all potential issues regarding sampling, sample storage, sequencing, and data analysis. It is crucial to realize that MAS should be considered a tool for assisting with traditional selection, not as a replacement for it [72].

It is not simple to improve beef quality traits. Many of them cannot be measured directly [33], and measuring them is subjective (as it is often done by a sensory panel, thus it may depend on the personal preferences of testers), is expensive, and is possible only after slaughter [72]. Thus, the modern beef industry is seeking biological factors (e.g., molecular markers) or biochemical methods for assessing beef quality while the animal is still alive. Furthermore, classification based on a subjective, visual characterization by a panel of specialized testers is being abandoned for more objective methods, which are automated, non-destructive, non-invasive, and cost-effective [69]. There are currently three main challenges facing the beef industry with regard to the use of genetic selection markers: i) the adequate collection of DNA marker information by breed association, ii) quality control mechanisms for the use and interpretation of DNA marker information, and iii) increased expertise in using the genetic markers discovered by the scientific community. The main goal of genetic improvement should be an adaptation of farming practices to single animal capacities [73].

Another issue besides the ‘better genetics’ of a single animal is that of consumer satisfaction. There is increasing emphasis on the developing suitable and efficient animal production systems due to the challenges of global climate change and the need to produce animal products that meet consumers’ needs (e.g., diet, health, the quality of products of animal origin, as well as animal welfare and the ethics of meat production). Consumers’ decisions about buying beef are based on an assessment of the meat’s tenderness/juiciness (which depends on genetic factors), as well as taste preferences. The whole carcass quality prediction system may be not sufficient to accurately predict the palatability of cooked beef. Furthermore, red meat is considered less healthy than other types of meat, thus enhancing its nutritional value, e.g., by increasing its omega-3 FA content, could be very beneficial from the customer’s point of view. Moreover, the awareness of consumers regarding animal welfare [74] and the environment continues to increase [75]. The animal production sector is considered one of the main contributors to climate change. Greenhouse gas emissions per unit of product could be decreased by improving the efficiency of the livestock production systems themselves or by targeting the source of the emissions, e.g., by using novel feeding technologies to reduce ruminants’ methane emissions [68,76]. Thus, breeding programs focused on increased efficiency could positively contribute to the climate change problem. In summary, consumers demand meat of good quality, but animal welfare and protection of the natural environment have become essential aspects of animal breeding; thus, meat palatability and welfare during rearing and at slaughter need to be addressed simultaneously [69].

## SUMMARY

In this review, we discuss selected SNPs within particular genes and their effects on beef quality (Table 1, 2). The single nucleotide changes in genes may affect protein sequence, and thus alters its structure and changes its functionalities or interactions with other proteins. Leptin has been identified as being mainly responsible for carcass fat and growth rate. It has been shown that various SNPs in this gene can alter the meat quality, e.g., UASMS2—affects the meat flavor, most probably due to the higher fat content and better MBS or C1180T—affects marbling and fat content. None of the leptin SNPs affect intramuscular fat (e.g., UASMS1, UASMS2). However, some studies failed to prove SNPs associations with analyzed meat traits (e.g., Y_11369.1:g.1620G>A). The TG regulates metabolism, fat deposition, fat cell development. SNPs in this gene may affect marbling (e.g., T354C, G392A, A430G, and A506C) or total lipids (e.g., C422T). Moreover, SNPs in calpain and their inhibitor calpastatin responsible for meat aging post-mortem affect many meat traits and alter meat tenderness (e.g., calpain: c.947G>C, 6546C>T, or calpastatin: A2959G). Yet, some studies fail to prove SNPs’ association in the calpastatin gene with beef quality traits (e.g., T596C, g.282C>G). Changes in the sequence of another gene, coding stearoyl-CoA desaturase, which influences saturation of FAs, introduce changes in the FA composition of meat (e.g., T878C, g.4706C>T, g.7864C>T affects MUFA percentage in intramuscular fat) or affects MBS (e.g., g.7534G>A). Another promising candidate SNP seems to be diacylglycerol acetyltransferase affecting general fat content in meat (e.g., c.572A>G SNP, c.1416T>G, K232A) measured as backfat thickness, MBS, fat color, meat tenderness or sirloin weight, and sirloin fat depth.

The inconsistency in the results mentioned in the manuscript sections dedicated for each gene may arise from the improper study design (the experimental group being too small; further study needed) and different genetic background of animals—divergent breeds and populations. Moreover, the overall effect of the particular SNP in some cases may be strongly related to cow species or cow breed (e.g., TG: C422T – MBS only in Wagyu, CAPN1 A4558G – generally affects tenderness and marbling, except for Chinese Simmental, CAPN 6546C>T – mainly increase meat tenderness, except for European cattle) and therefore association study may also fail due to high variability in experimental groups. The exert effect of SNP often depends on the predominant function of protein coded by gene, in which SNP is present—SNPs in leptin or TG affects MBS or fat content, changes in calpain or calpastatin amino acids sequence affects general meat tenderness, or stearoyl-CoA desaturase SNPs affecting FA content—changing MUFA, SFA and particular FAs concentrations (e.g., T878C SNP, g.4706C>T, g.7534G>A, g.7864C>T). Some SNPs may have some additional effects, such as changes in calpain sequence may alter meat tenderness and flavor or juiciness (e.g., A2959G). SNPs in diacylglycerol acetyltransferase, besides FAs content, may also change MBS or fat color (e.g., c.572A>G, c.1416T>G). This summary shows that despite the vast potential of SNP implementation in breeding programs for meat quality improvement, each gene should be considered as part of multiple metabolic pathways, part of a network, and altering it may lead to further changes that may or may not cause the intended effect. This review has a huge potential to serve as a guideline or starting point for people interested in MAS implementation in animal breeding. It may help to increase the prevalence of favorable allele frequencies in animal populations and, therefore, significantly improve the quality of produced beef.

## CONCLUSION

Although the production traits described above are under polygenic regulation, it is crucial to apply those markers in breeding programs to obtain animals with better genetics. Meat quality traits are also influenced by the conditions under which animals are kept. Animals with better genetics will exhibit desired properties aiming meat quality traits. Placing animals with exceptional genetic potential in the right environment, with proper keeping and feeding conditions, may produce meat of exceptional quality. It is vital to develop methods that simultaneously improve beef quality and safety, decrease the carbon footprint of meat production, and consider animal welfare. Science and innovation may introduce breeders and the meat industry to new tools and technologies that respond to consumers’ concerns and expectations.

## Figures and Tables

**Table 1:** Description of genes and single nucleotide polymorphisms associated with beef traits

Gene	SNP	Meat trait	Function	References
*lep*	UASMS2	Meat flavor	Animals with TT genotype having higher liking score than animals with CT or CC genotypes	[16]
		Sirloin fat thickness	Animals with CC genotype having less fat surrounding the sirloin than animals with CT or TT genotypes	
		Backfat thickness, marbling score	Animals with TT genotype having a higher value for backfat thickness and marbling score than animals with CT or CC genotypes	[20]
	C1180T	Backfat thickness, marbling score	Animals with CC and CT genotypes having a higher value for backfat thickness and marbling than animals with TT genotype	[22]
	Exon 2, arginine to cytosine substitution	Carcass grade fat	Animals with TT genotype having more carcass grade fat than animals	[23,24]
		Meat yield	Animals with TT genotype having lower meat yield	
*TG*	C422T	Marbling score	Animals with TT genotypes having higher MBS than those with CC and CT genotypes	[32]
			Animals with CC and CT genotypes having higher MBS than animals with TT genotypes	[27]
	G133C	Marbling score	Animals with CC genotype having higher values of marbling score than animals with GG and GC genotypes	[28]
	T354C			
	G392A			
	A430G			
	T433G	Marbling score	Animals with CC genotype having higher value of MBS than animals with CT or TT genotypes	[34]
	X05380.1:g.-422C>T	Meat tenderness	Animals with CT genotype having more tender meat than homozygotic animals with CC and TT genotypes	[35]
*CAPN1*	c.947G>C	Meat tenderness	Animals with CC genotype having more tender meat than animals with GG genotype (the heterozygote produced meat with a tenderness score between that of homozygotes)	[1]
	C6545T	Meat tenderness	Animals with CC and CT genotypes having more tender meat than animals with TT genotype (no differences were observed in the Brahman)	[39]
		Meat flavor	Animals with CC and CT genotypes having meat with a more intense flavor when compared with animals with TT genotype	
	A4558G	Meat tenderness	Animals with GG genotype having lower values	[42]
		Marbling score	Animals with GG genotype having lower values	
	C4684T	Meat tenderness	Animals with CC genotype having a higher value than animals with TT genotypes, and animals with CT genotypes having higher values than animals with TT genotypes	
	c.2151*			
	479C>T	Meat tenderness	Animals with CC genotype having higher values	[43]
	capnI/BtgI	Meat tenderness	Animals with GG genotype having higher values	[44]
	capn4751	Meat tenderness	Animals with TT genotype having higher values for shear force, and animals with CT genotype having higher values for myofibrillar fragmentation index	[46]
	g.5709C>G	Meat tenderness	Animals with GG genotype having higher values for shear force and myofibrillar fragmentation index	[47]
*CAST*	A2959G	Meat tenderness	Animals with AG and AA genotypes with higher values for shear force and myofibrillar fragmentation index	[46]
			Homozygotic animals having a lower value for shear force than heterozygotic animals	[48]
		Juiciness	Homozygotic animals having a lower value for shear force than heterozygotic animals	
		Meat flavor	Homozygotic animals having a lower value for shear force than heterozygotic animals	
	g.98535683A	Meat tenderness	Animals with AA genotype having more favorable phenotype than animals with AG genotypes	[49]
*SCD*	T878C	Fatty acid composition	Higher MUFA percentage in intramuscular fat	[50]
		Fatty acid composition	Animals with AA genotype having a lower content of C18:0 and SFA compared to animals with VV genotypes; animals with AA genotypes having more C14:1 cis-9 compared to animals with AV and VV genotypes; the content of C18:1 cis-9 was higher in AA and AV compared to VV; the bulls with genotypes AA or AV had lower SFA, higher MUFA, and higher MUFA/SFA than the VV animals; animals with the AA and AV genotypes having higher values for C14, C18, and total desaturation than VV	[51]
		Fatty acid composition	Animals with CC genotypes with lower concentrations of SFAs, higher concentrations of MUFAs, and a higher concentration of polyunsaturated fatty acids compared to TT animals	[52]
		Intramuscular fat, meat tenderness	Homozygotic animals having a higher value than those with heterozygotic animals for both traits	[54]
		Fatty acid composition	Homozygotic animals with AA genotype having the highest content of MUFA than animals with VA genotype, the lowest MUFA content for animals having VV genotype	[50]
	g.4706C>T	Fatty acid composition	Animals with CC genotype having the lowest relative amount of SFA, followed by animals with CT and TT genotypes, respectively; animals with CC genotype having a higher relative amount of MUFA than animals with TT genotypes	[55]
	g.7534G>A	Marbling score	Animals with GG and GA genotypes having a higher value of MBS than AA animals	
		Fatty acid composition	Animals with GG genotype having a lower relative amount of SFA than animals with AA genotype	
	g.7864C>T	Fatty acid composition	Animals with CC genotypes having higher values for the relative amounts of SFAs and MUFAs, than animals with TT genotype	
*DGAT1*	c.572A>G	Backfat thickness, marbling score, fat color, meat tenderness	Animals with the BB genotype having higher values for backfat thickness and lower values for MBS, fat color, and shear force than animals with AA genotype	[56]
	c.1416T>G	Backfat thickness, marbling score, fat color, meat tenderness	Animals with FF genotypes having higher values for backfat thickness and lower for fat color, and shear force than animals with EE genotype	
*GH*	c.457C>G	Fat thickness	Animals with CC genotype having a higher value for fat thickness	[59]
	L127V	Fatty acid composition	When allele L was substituted for allele V, the proportion of C16 or shorter fatty acids (C14:0, C14:1, C16:0, C16:1) decreasing while the proportion of C18:1 increasing	[11]
		Subcutaneous fat thickness	A significant effect of GHL127V on subcutaneous fat thickness was detected	
	T172M	Carcass quality	Gene effects of GHT172M were detected for all analyzed types of carcass traits – carcass weight, rib thickness, subcutaneous fat thickness, and firmness	
*FABP4*	c.220A>G (I74V)	Fatty acid composition	Animals with II genotype having a higher percentage of C16:1 than animals having VV genotype	[61]
		Fatty acid composition	Animals with VV genotype having a higher percentage of C10:0 than animals with II genotype	[62]
		Backfat thickness	Heterozygotic animals having a higher value than homozygotic animals	[63]
	c.328G>A (V110M)	Carcass weight	Heterozygotic animals having a higher value than homozygotic animals	
	c.280A>G	Backfat thickness	Animals with GG genotype having lower values for backfat thickens than AA and AG animals	[10]
		Fatty acid composition	Animals with GG genotype having a higher content of C18:1 than AA and AG animals; homozygotic animals with GG genotype having a higher content of C18:2n6 than AA and AG animals; homozygotic animals with GG genotype having lower SFA content than AA and AG animals; homozygotic animals with GG genotype having higher MUFA content of than AA and AG animals	
	c.388G>A	Marbling score	Animals with AA genotypes having lower values than animals with GG and AG genotypes	
		Fatty acid composition	Animals with AA genotype having a lower content of C18:1 than GA and GG animals; homozygotic animals with GG genotype having higher MUFA content than GA and AA animals	
	c.408G>C	Fatty acid composition	Animals with CC genotype having a higher content of C16:0 than animals with GG and GC genotypes; animals with CC genotypes having a higher content of C18:1 than animals with GG and GC genotypes; animals with CC genotypes having a lower content of C18:2n6 than animals with GG and GC genotypes; animals with CC genotype having lower SFA content than animals with GG and GC genotypes; animals with CC genotypes having higher MUFA content than animals with GG and GC genotypes	
	c.456A>G	Fatty acid composition	Animals with GG genotype having a higher content of C18:1 than animals with AA and AG genotypes; animals with GG genotype having a lower content of C18:2n6 than animals with AA and AG genotypes; animals with GG genotype having lower SFA content than animals with AA and AG genotypes; animals with GG genotype having higher MUFA content than animals with AA and AG genotypes	
	g.7516G>C	Marbling score, subcutaneous fat depth	Animals with GG genotype having a higher value than animals with GC and CC genotypes for both traits	[64]
*FASN*	g.17924A>G	Fatty acid composition	Animals with AA genotype having higher concentrations of SFA 14:0 and a lower concentration of oleic acid	[52]
		Fatty acid composition	Homozygotic animals with GG genotype having lower c14:0 level, c16:0 level, total SFA content, and higher health index, c18:1 content, total MUFA concentration in the total lipids and triacylglycerol fraction than animals with AA genotype	[12]

SNP, single nucleotide polymorphism; *TG*, thyroglobulin; MBS, marbling score ; *CAPN1*, calpain 1; *CAST*, calpastatin; *SCD*, stearoyl-CoA desaturase; MUFA, monounsaturated fatty acid; SFA, saturated fatty acids; *GH*, growth hormone; *DGAT1*, diacylglycerol acetyltransferase; *FABP4*, fatty acid-binding protein 4; *FASN*, fatty acid synthase.

**Table 2:** Selected meat traits and associated single nucleotide polymorphisms

Trait	Gene	SNPs	References
Marbling score	Leptin	UASMS2, C1180T	[20,22]
	Thyroglobulin	C422T, G133C, T354C, G392A, A430G, T433G	[27,28,32,34]
	Calpain	A4558G	[42]
	Stearoyl-CoA desaturase	g.7534G>A	[55]
	Diacylglycerol acetyltransferase	c.572A>G, c.1416T>G	[56]
	Fatty acid binding protein 4	c.388G>A, g.7516G>C	[10,64]
Meat tenderness	Thyroglobulin	X05380.1:g-422C>T	[35]
	Calpain	c.947G>C, C6545T, A4558G, C4684T, c.2151*479C>T, capnI/BtgI, capn4751, g.5709C>G	[1,39,42,43,44,46,47]
	Calpastatin	A2959G, g.98535683A	[46,48,49]
	Stearoyl-CoA desaturase	T878C	[54]
	Diacylglycerol acetyltransferase	c.572A>G, c.1416T>G	[56]
Fatty acid composition	Stearoyl-CoA desaturase	T878C, g.4706C>T, g.7534G>A, g.7864C>T	[50–52,55]
	Growth hormone	L127V	[11]
	Fatty acid binding protein 4	c.220A>G, c.280A>G, c.388G>A, c.408G>C, c.456A>G	[10,61,62]
	Fatty acid synthase	g.17924A>G, g.17924A>G	[12,52]
Backfat thickness	Leptin	UASMS2, C1180T	[20,22]
	Diacylglycerol acetyltransferase	c.572A>G, c.1416T>G	[56]
	Fatty acid binding protein 4	c.220A>G, c.280A>G	[10,63]
Meat flavor	Leptin	UASMS2	[16]
	Calpain	C6545T	[39]
	Calpastatin	A2959G	[47]
Sirloin fat thickness	Leptin	UASMS2	[16]
Rib thickness	Growth hormone	T172M	[11]
Subcutaneous fat thickness	Growth hormone	L127V, T172M	[11]
Carcass grade fat	Leptin	Exon 2, arginine to cytosine substitution	[23,24]
Meat yield	Leptin	Exon 2, arginine to cytosine substitution	[23,24]
Carcass weight	Growth hormone	T172M	[11]
	Fatty acid binding protein 4	c.328G>A	[63]
Firmness	Growth hormone	T172M	[11]
Juiciness	Calpastatin	A2959G	[48]
Intramuscular fat content	Stearoyl-CoA desaturase	T878C	[50,54]
Fat deposition	Diacylglycerol acetyltransferase	K232A	[16]
Fat color	Diacylglycerol acetyltransferase	c.572A>G, c.1416T>G	[56]
Fat thickness	Growth hormone	c.457C>G	[59]

SNPs, single nucleotide polymorphisms.
